# Technostress and Employee Performance Nexus During COVID-19: Training and Creative Self-Efficacy as Moderators

**DOI:** 10.3389/fpsyg.2021.595119

**Published:** 2021-10-13

**Authors:** Farida Saleem, Muhammad Imran Malik, Saiqa Saddiqa Qureshi, Muhammad Faisal Farid, Sabeen Qamar

**Affiliations:** ^1^Department of Management, College of Business Administration, Prince Sultan University, Riyadh, Saudi Arabia; ^2^Department of Management Science, COMSATS University Islamabad, Attock, Pakistan; ^3^Department of Business Administration, Fatima Jinnah Women University, Rawalpindi, Pakistan; ^4^Department of STEM Education, University of Education, Lahore, Lahore, Pakistan; ^5^Institute of Education and Research (IER), University of the Punjab, Lahore, Pakistan

**Keywords:** COVID-19, techno-stress, creative self-efficacy, employee performance, training, universities, social exchange theory

## Abstract

Technostress, a stressor, has implications for employee’s psychological states; however, flexibility like work from home can have positive outcomes, especially for instructors who have to teach and ensure social distance during COVID-19. The present study examined the relationship of technostress and employee performance while taking training and creative self-efficacy as boundary conditions. A sample of 222 university instructors, who worked from home or hybrid (home and workplace) during COVID-19 lockdown, was selected from Pakistan. The responses were recorded using a closed-ended questionnaire. Stepwise linear regression and PROCESS Macro by Hayes (2013) was used to analyze the data. It was revealed that technostress, instead of having adverse effects, had a positive effect on employee’s performance and both training and one’s creative self-efficacy significantly moderated the relationship. As the main finding, it was revealed that the employees continued to perform well despite the prevalence of technostress. The training and one’s creative self-efficacy were useful to control the technostress and maintain the performance of instructors during COVID-19. The university administrators and employees must take technology as a positive tool for performance. The training, along with creative self-efficacy, adds to the working capacity of employees and enhances their performance.

## Introduction

Consistent employee performance brings the organization’s effectiveness (Shet et al., 2019). However, it becomes a great challenge to manage employee performance during a constantly changing environment (Rodrigues and Pinho, 2012). The external environmental changes, like COVID-19, have urged people to adopt technological solutions. However, not always, these solutions lead to productive outcomes and may result in inducing technology overload and anxiety (Atmaja et al., 2018), and employees’ performance may decline. The anxiety and complex use of technology pave the way to technostress (McFedries, 2003; Maier et al., 2019), and it may shatter the employee’s confidence to work. It may also induce withdrawal behaviors and hampers employee performance (Yang et al., 2017). The COVID-19 has brought threats to the individuals’ health and pushed them to remain at a distance from each other. Remaining at a distance (social distancing) and work from home to meet the work demands has compelled employees to adopt information and communication technology (ICT) as an appropriate tool. These abrupt demands related to technology adoption by learning new skills stimulate technostress among employees (La Torre et al., 2019).

The technological revolution has introduced several positive changes to work practices. It can foster work pace and bring efficiency (Tarafdar et al., 2015) and is also associated with various work attitudes and behaviors (Yang et al., 2017). Adopting technology is not always easy (Prabhakaran and Mishr, 2012), and the stress related to adopting new technology establishes a negative psychological link between the individual and the new technologies. Technostress is defined as “a negative psychological state associated with the use or the “threat” to use new technologies,” which leads to “anxiety, mental fatigue, skepticism, and sense of ineffectiveness” (Salanova et al., 2013). Prior research on technostress has primarily suggested that technostress is damaging and can have harmful impacts on employees’ work performance (Tarafdar et al., 2007, 2010). The negative impact of technostress on employees’ productivity is easily comprehendible. However, we do have empirical findings available supporting either significant positive or insignificant impact of technostress on employees’ productivity (Tu et al., 2005, 2008; Hung et al., 2011). Due to the equivocal findings related to technostress and employee performance nexus, two boundary conditions are proposed that make the relationship significantly positive. These boundary conditions, employee training, and their creative self-efficacy act as a dual process.

The stress due to technology adoption may result in a slow work pace of employees, declined motivation to work, lower levels of organizational commitment by disturbing their work-life balance, and declined performance (Raišienë and Jonušauskas, 2013). However, the performance of employees’ can be preserved by maintaining social exchanges in the organizations like providing training to the employees and admiring their creative efforts to perform better. The training about how to use technology enables the employees to perform better and achieve their targets. As the technology can be used as a tool to manage work (Wolor et al., 2020), the employees trained for how to use technology are likely to perform better than untrained ones (Holman et al., 2020).

With this study, it is posited that the social exchange theory (Ekeh, 1974; Emerson, 1976) and transactional model of stress and coping developed by Lazarus and Folkman (1987) provides an alternative explanation for describing employees’ performance through various factors. This study has proposed training and creative self-efficacy as the mechanisms that help to reduce the adverse effects of technostress and enhance performance. It is argued that an individual’s creative self-efficacy brings enhanced motivation to do work, induces higher organizational commitment, and results in better performance. In organizations, the individuals interact and develop relationships that are strengthened by developing a sense of trust and reciprocity. This trust and sense of reciprocity motivate employees to perform better in the organization to fulfill the organizational goals (Cook et al., 2013). The current investigation propose that such exchanges can potentially reduce the levels of stress and enhance motivation, and gives confidence to employees to perform better. The phenomenon of technostress is emerging and not yet thoroughly examined (Tarafdar et al., 2015). Earlier studies have examined different segments of the economy and have ignored the education sector that is considered by the current study. The excessive demands to use technology due to COVID-19 have sparked out a new dimension of stress to be explored. The current investigation has proposed a new framework involving the social exchange theory for the employees’ performance. It is posited that employees’ performance is an organizational resource that can be enhanced through employee-employer contributions together. The training comes from the employer side and creative-efficacy from the employee’s side as sources of social exchange that enhance reciprocity among both.

This study contributes to the literature in many ways; first, the current study aims to analyze the relationship of technostress and performance among university instructors in times of COVID-19 that has possible effects on the performance of university instructors. The university management on one side demanded continued high performance from instructors, and on the other side, the COVID-19 lockdown demanded social distancing. Hence, the use of technology was considered a possible solution to meet both demands. In the present study, a multidimensional analysis of the phenomenon was carried out in which the relationship of subjective technostress and job performance was analyzed. However, The existing literature on information systems (IS) and stress was mainly related to IS managers or employees in technology companies, and the “technology stress” was framed as a negative phenomenon (e.g., Tarafdar et al., 2015; Harris et al., 2021). Secondly, this study is different in context compared to earlier investigations. The outbreak of COVID-19 demanded excessive use of technology for maintaining social distancing during instructor-student interactions and learning processes. Employees’ work performance determines why some organizations outperform others. The importance of work performance also applies to higher education settings where there have been increasingly intense competitions among universities worldwide over students, funding support, and reputations (Wæraas and Solbakk, 2009).

The current study helps university administration to understand factors affecting university instructors’ work performance during COVID-19. Thirdly, with the current investigation, also aim to extend the literature on employee performance by analyzing the moderating role of training and creative self-efficacy in the relationship between technostress and employee performance. The current investigation contribute to the literature by enhancing the current understanding of the relationships through the lens of social exchange theory, wherein the positive reciprocal relationships support the employees to perform well and by presenting training and creative self-efficacy as coping strategies. Lastly, the study has been conducted in a developing country, Pakistan, where the limited technical facilities and IT infrastructure may provide differing results regarding the behaviors of the employees working in universities during COVID-19.

## Theoretical Framework and Hypotheses Development

### Social Exchange Theory and Transactional Model of Stress and Coping

According to social exchange theory (SET), various stakeholders may be involved in the exchange of resources in an organization (Blau, 1968). These stakeholders develop relations so that they feel responsible for reciprocating in the same manner in which their organization has treated them. At the same time, the relationships of trust are developed over time, and employees want to maintain the relationships of trust and reciprocity (Cropanzano and Mitchell, 2005). This relationship signifies reciprocity, where one party may reciprocate the other by returning the favor. Hence, if an organization provides support and benefits like the training to equip its employees with the latest knowledge and skills, the receiving individuals may return the benefit by being more committed and by showing an outstanding performance (Gergen, 1977). When employees get something from their organization as support, they try to return by contributing more toward the organization. Based on SET, it is proposed that when employees are provided with the training for the use of technology, it enhances their performance and reduces technostress. Employees consider this training as a resource that provides the support for better functioning. In return, using their capabilities, the employees perform their best to meet the organizational goals and maintain their competitive position.

Moreover, the support from the organizations adds to the self-efficacy of employees (Caesens and Stinglhamber, 2014). The low productivity of employees due to weak self-efficacy threatens the organization’s competitiveness and sustainability (Bandura, 2000). At the same time, employees with higher self-efficacy feel satisfied and concentrate on their work roles. They try to use more innovative ways to perform and get back to their managers with positive input (Asgari et al., 2020). The support from the organization help in enhancing the creative self-efficacy of employees, which in return triggers reciprocity behavior from employees in the form of enhanced performance levels.

Based on these arguments, this study adopts SET to understand how training and creative self-efficacy interact to affect technostress and, ultimately, employee performance. Training and creative self-efficacy has been presented as the dual process that acts as boundary conditions for the relationship of technostress and employee performance. It is assumed that overall stress is decreased with the provision of training (Weinert et al., 2020) to use technology, and additionally, one’s creative self-efficacy helps in learning, thus supporting their performance.

According to transactional theory of stress (TTS), the perceived imbalance between the demands of a person’s environment and the available resources the person possesses to respond to the results into stress (Lazarus and Launier, 1978; Lazarus and Folkman, 1984; Lazarus, 2006). The transactional model of stress and coping developed by Lazarus and Folkman (1984) explained coping as a phenomenon involving cognitive and behavioral responses that individuals use to manage internal and/or external stressors perceived to exceed their personal resources. According to Weinert et al. (2019), coping is a function of an individual’s cognitive, behavioral, and perceptual efforts to control stressful situations. These strategies can be proactive or reactive, depending upon an individual’s coping mechanism. Lazarus and Folkman (1984) model of stress and coping has five components: person and environment influencing factors, cognitive appraisals, stress, coping response, and adaptational outcomes. Lazarus and Folkman (1984) defined the primary appraisal as “the judgment that an encounter is irrelevant, benign-positive, or stressful” and secondary appraisal as “a judgment concerning what might and can be done” (Lazarus and Folkman, 1984, p. 53). The secondary appraisal is related to the coping response and strategies to deal with stressful situations. It is the human tendency to look for alternatives to resolve problems.

According to Cooper et al. (2001) “stress is an ongoing process that involves individuals transacting with their environment.” (2001; pp. 12). The holistic stress process has four stages (environmental conditions, stressors, psychological responses and outcomes) connected with each other with three evolution processes (appraisal process, decision process, and performance process) (McGrath, 1976). Regarding technostress, the transactional model of stress (TTS) identifies the stress appraisal and coping mechanism as determining processes for the influence of techno stressor (Al-Fudail and Mellar, 2008; Ragu-Nathan et al., 2008). In connection with technology and ICT use, stress can result from a difference in the actual technology competency level and competence requirements, thereby threatening user well-being (Fuglseth and Sørebø, 2014). Technostress as a stressor is expected to increase strain for the employees and bring low motivation to work and dissatisfaction. When the employees are faced with overload, complexity, uncertainty, etc., (as creators of stress), they become overwhelmed and lose concentration on work, thus resulting in low performance. The transactional model of stress and coping identifies that the primary appraisal of the situation will result in the identification of stress, and the secondary appraisal will trigger the coping mechanism. Zhao et al. (2020) have used TTS while focusing on the appraisal and coping processes and empirically investigated these two processes of technostress and have identified coping strategies as an important mediating process. The coping strategies as a process can be triggered from inside, in the form of the creative self-efficacy of an individual. Similarly, it is propose that the coping strategies can be triggered from outside in the form of training and development provided by organizations. These two variables, “training” and self-efficacy,“ act as coping strategies to stress that help enhances performance.

**FIGURE 1:**
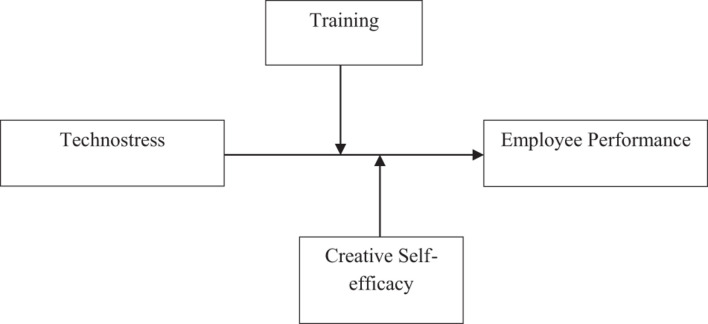
Research framework.

Based on SET and the transactional model of stress and coping, it is proposed that employees try to reciprocate the exchanges made within organizations and try to resolve the stressful situations (technostress) by focusing on developing coping strategies (secondary appraisal or psychological response). In the light of the said argument, it can be suggested that the transactional model of stress and coping is embedded in the proposed theoretical model where the current investigation has presented training and creative self-efficacy as coping strategies. Hence based on the above discussion, the proposed research framework is presented in Figure 1.

### Technostress and Employee Performance

With the outbreak of the COVID-19 pandemic, technology has gone excessive, influencing all aspects of employee attitudes and behaviors at the workplace. However, employee performance is the key to organizational success (Rodrigues da Costa and Maria Correia Loureiro, 2019). The pressures to use technology may harm employee performance. The organizational demands to adopt technology can result in technostress generation (La Torre et al., 2019). This stress brings physical and emotional exhaustion among employees (Raišienë and Jonušauskas, 2013) and negatively affects their performance. The information systems research on stress, known as research on technostress, has found that the technology induces harmful psychological stress and is concomitant with adverse organizational outcomes (Tarafdar et al., 2010, 2015; Ayyagari et al., 2011). Prior studies noted that for university instructors, the integration of ICT into classrooms results in work overload, role ambiguities, changed patterns of work, needs for constant upgrade of knowledge and skills, and higher demands for performance and productivity (Tarafdar et al., 2010; Jena et al., 2015).

Work stress is seen as a mismatch between the job requirements and an individual’s ability to cope with those demands (Yang et al., 2017), including effective adoption and use of technology. The adoption of new technology may result in work overload (Ahmad and Scott, 2019). This work overload may reduce employees’ job satisfaction and compel them to develop intentions to leave the organization (Holland et al., 2019), thus contributing negatively to their job. The technology requires employees to work faster to keep track of the organizational goals (Atmaja et al., 2018). The accelerated pace of work may develop time pressures upon employees. They feel overwhelmed (McFedries, 2003), thus losing concentration on work, thus doing work quickly. Moreover, it is merely possible that the employees who have to learn the new knowledge and skills may spend much time learning the new technology (Gardner et al., 2019), and the time they needed to do their job may be lost, thus hampering their performance.

Similarly, a lack of clarity about the procedures may also add stress to the employees, which may create work ambiguity (Frögéli et al., 2019). Employees’ efforts to find solutions to the problems they face may also distract their concentration from work, and they may not perform as per requirements (Biron and Boon, 2013). The organization’s job demands (use of technology) can influence employee’s commitment levels, job satisfaction, and ultimately their performance (Han et al., 2020). However, literature has also provided support for technostress as eustress. Raftar (1998) empirically established a significant positive impact of technostress on job performance. According to Raftar (1998), the proactive approach to technology acceptance and utilization is associated with enhanced performance. Similarly, Hung et al. (2011) have also found support for a positive impact of technostress on productivity. Hence, based on the above discussion, it is proposed that.

*Hypothesis 1: Technostress will have a significant impact on employee’s work performance.*

### Training (as a Moderator) and Employee Performance

As a means of social exchange, the training can improve the employee’s performance (Ji et al., 2012). Organizations can enhance employee competitiveness by improving employee skills, knowledge, and abilities (KSA’s). The enhancement in employee’s knowledge and skills results in human capital development to tackle any situation occurring in the workplace that further improves organizational performance (Schraeder, 2009). Training improves individual capabilities, such as coping with stress. The technostress is the mismatch between the individual capacities and technological demands (Pirkkalainen et al., 2019). The higher the mismatch level, the higher the produced stress (Ahmad et al., 2009; Prabhakaran and Mishr, 2012). This stress reduces the work potential and diminishes the workplace (Vischer, 2007).

The training is considered an effective stress coping strategy (Skinner et al., 2003; Salo et al., 2020). Training instills the capability to handle the challenges arising out of work. Training based on handling unavoidable situations such as COVID-19 and the use of technology to perform work can be helpful in complex work environments (Li et al., 2017) and enhance the levels of motivation to adapt according to the situations. On the other hand, the organization’s failure to provide relevant training may result in unwanted work outcomes (Athar and Shah, 2015), like the inability to perform as per requirements to meet the organizational goals. It is also noted that the training enables employees to maintain their commitment to their work and organization (Elnaga and Imran, 2013), enhances their control over work, and results in better work outcomes. The higher the work control achieved through using the tools and techniques available, the less technostress is likely to occur (Brivio et al., 2018). Thus, it is argued that employee training may have a positive effect on reducing overall stress. The best use of the knowledge, skills, and abilities does not let employees overwhelmed by work (Birdi et al., 2008). Based on the above arguments, the following hypothesis is proposed.

*Hypothesis 2: The training moderates the relationship between technostress and an employee’s work performance.*

### Creative Self-Efficacy (as a Moderator) and Employee Performance

General self-efficacy is frequently studied in relation to employee performance (Bandura, 1986; Hayashi et al., 2004; Alghamdi et al., 2020). Closely related to general self-efficacy, creative self-efficacy (CE) is defined as the belief that one can produce creative outcomes (Tierney and Farmer, 2002; Tierney and Farmer, 2011). The creative self-efficacy, or the creative efficacy, may reinforce self-efficacious employees to put in extra effort to learn and perform (Tierney and Farmer, 2011). It makes them highly motivated to achieve the extra mile for effectiveness. The efficacious creative employees are found willing to exchange knowledge and skills to develop unique ideas and easily find a solution to the problems (Farmer and Tierney, 2017). These unique ideas and alternatives to solve problems help them remain engaged in work (Ismail et al., 2019) and perform to achieve their targets.

As a case of reciprocity, the creative employees tend to achieve organizational effectiveness and are considered essential for the organization (Hughes et al., 2018). The individuals with enriched CE are likely to share creative ideas and suggest solutions to the problems, thus paving the way for creating a positive work environment that supports themselves and their colleagues (Jaussi et al., 2007; Kremer et al., 2019). The high CE people are involved in innovative behaviors because of their confidence (Hughes et al., 2018). They are more involved in knowledge seeking and sharing, thus continuously updating the job-related knowledge (Hu and Zhao, 2016; Cheng, 2019). Therefore, they are more likely to overcome the problems of technology use and perform up to satisfaction levels. The above arguments helped in devising the hypothesis such as;

*Hypothesis 3: Creative self-efficacy moderates the relationship between technostress and employee performance.*

## Methodology

### Sample and Data Collection

A sample of male and female instructors was considered for the study from the public and private sector universities. The universities were selected because the educational institutions have undergone a massive transition to avoid the harms of COVID-19. The universities had shifted to adopt new technologies using various platforms for interacting with students and university administration. They used various tools for interaction such as zoom, Google classroom, Skype, MS teams, etc., The adoption and use of these tools engulfed people with technostress as most were not used to using them in their everyday lives. The employees, especially instructors, have either learned these tools through the formal training provided by their universities or on their own, using various sources.

The questionnaire used for data collection was presented to the COMSATS University Islamabad, Attock Campus, Pakistan ethical committee and was approved. A sample was selected from public and private sector universities to know the difference between the groups. The public sector universities are likely to have people-oriented management compared to the private sector universities having task-oriented management, which is likely to affect their work performance. Moreover, the responses were gathered from various departments. The people who are less used to interaction with technology may likely develop technostress quickly, and thus, their performance may decline.

Prior permission was sought from the university administration to gather the responses for a research study. On the day of a visit, all precautions were taken to avoid any possible threat of COVID-19. While entering the university, the university staff examined the temperature before entering the premises. During the partial lockdown, very few and selected employees/instructors were allowed to visit the university. Thus, the contact information of other teaching staff was sought through using referrals (snow-ball sampling technique). The people were approached through their emails, and a request was made for filling out the questionnaire.

### Instrumentation

A closed-ended, self-report questionnaire was used to gather responses. The questionnaire was divided into two sections the first section was regarding the demographic information of respondents, and the second section was related to the variables present in the proposed model. All items presented in the scale except demographic information of respondents were measured on five points Likert scale where 1 was “strongly disagree” to 5 as “strongly agree.”

#### Technostress

The technostress was measured using a questionnaire adapted from Tarafdar et al. (2007), and minor modifications were introduced to meet the requirements of the study. These minor modifications were used in defining the term technology as “computer-related technologies used for teaching and other work -elated purposes.” It comprises the fourteen statements with three underlying dimensions, including techno-overload, techno-complexity, and techno-invasion. The sample statements included were, for example, “I am forced by technology to work much faster,” “I am forced by technology to do more work than I can handle,” and “I am forced by the technology to work with very tight time schedules.” The Cronbach’s alpha for the scale was 0.946.

#### Creative Self-Efficacy

The creative self-efficacy scale was adopted from Brockhus et al. (2014), having seven items, minor modifications were introduced in the phrasing of two items where “friends” was replaced with “colleagues” and “creative idea” was replaced “creative solutions.” The sample items included were as; “I am a creative person,” “Compared to my colleagues, my ideas are outstanding,” and “I am confident that I can develop creative solutions for almost any problem.” The reliability alpha for the scale was 0.955.

#### Training

A questionnaire for assessing the effects of training was adapted from the study of Aziz (2015). Seven items were used of this scale. The sample items included were, for example, “I know how to solve certain job problems related to technology using the skills learned through training,” and “My personal competencies have improved after attending the trainings related to technology,” “I am being professional in certain tasks related to technology after getting training.” The Cronbach’s α score showed satisfactory reliability for the scale that was 0.950.

#### Employee Performance

Eight itemed contextual employee performance scale was adopted from Koopmans et al. (2014) for the measurement of performance of respondents. The sample items are “In the past three months I took on extra responsibilities.” and “In the past three months I took on challenging work tasks, when available.” It’s Cronbach’s α was 0.961.

### Common Method Variance and Social Desirability Bias

The common method variance and social desirability bias were controlled to affect the results (see for example; Podsakoff et al., 2003; Schwarz et al., 2017). The scales for measurement of each variable were either adopted or adapted since the weak questionnaire development is the main cause of non-response bias (Hill et al., 1997). Similarly, various methods were used to control the potential common method bias. Firstly, a prior consent from the respondents was requested. Moreover, the anonymity of the respondents was assured, along with maintaining the confidentiality of their responses, and they were not asked to write any personal identification information anywhere on the questionnaire. These steps helped reduce social desirability bias.

Additionally, the placement of the dependent and independent variables at separate positions was ensured in the questionnaire (Grimm, 2010). Placing the variables closely on the questionnaire could provide ques to the respondents by providing a common context (Podsakoff et al., 2003). Lastly, the issue of common method bias by using common latent factor analysis was statistically ruled out. As the cross-sectional data collection method was used, there is still the possibility of common method variance (Podsakoff et al., 2003). The common latent factor analysis was run, also known as the unmeasured latent method factor technique in AMOS, to identify common method bias. The results identified a 25% variance due to a common latent factor. The value is less than 50% cut-off value (Williams et al., 1989; Richardson et al., 2009). Hence there was no issue of common method bias.

## Data Analysis and Results

### Analytical Approach

A quantitative approach was adopted using primary data collected through a questionnaire survey. The final sample of 222 university teaching staff was used for data analysis. Stepwise linear regression and model no one and Model no 2 of PROCESS Macro by Hayes (2013) were used for data analysis. Out of 222 respondents, 74% of respondents were males. More than half of the respondents (53.1%) were in the age group of 36 to 45 years, followed by 36.0% of people who belonged to the age group between 26 to 36 years. 70.2% of employees had permanent jobs in the university, whereas around 30% provided their services as visiting faculty members. It is also noted that nearly half of the respondents were had MS or Ph.D. degrees (51%). Maximum of the respondents were had an experience of 6 to 10 years (44.5%), followed by the people having experience of 1 to 5 years (31.0%). Similarly, more than half of the respondents, 58%, were from private sector universities. 18.9% of respondents were teaching subjects of IT and computer and the majority were teaching other subjects in which the computers and information technology are not taught as a formal subject. The results of descriptive analysis of demographic variables are presented in Table 1.

**TABLE 1:** Demographic information, *n* = 222.

**Variables**	**Category**	**Frequency**	**Percentage**
Gender	Male	164	73.9
	Female	58	26.1
Age (years)	26–35	80	36.0
	36–45	118	53.1
	46–55	24	10.8
Job Status	Permanent	156	70.2
	Visiting	66	29.7
Education	Graduation	12	05.4
	Masters	97	43.6
	MS/Ph. D.	113	50.9
Experience (Years)	<1	15	06.7
	1–5	69	31.0
	6–10	99	44.5
	>11	39	17.5
Sector of University	Public	93	41.8
	Private	129	58.1
Discipline	Social Sciences	53	23.8
	Applied Sciences	38	17.1
	Humanities	89	40.0
	IT and Computer	42	18.9

*Source: Field Data.*

### Control Variables

To identify control variables and the significance of any demographic variable concerning the proposed model, one-way ANOVA test was used. None of the demographic variables was significantly associated with technostress, creative self-efficacy, training, and employee performance. Hence there was no need to control any demographic variable for further analysis. However, all demographic variables during stepwise linear regression analysis were controlled.

### Scale Validation

For the validation of the scale used for data collection, confirmatory factor analysis was used. Confirmatory factor analysis (CFA), also known as the measurement model, was conducted using AMOS 17. TS14 and EP7 were removed from further analysis due to its cross-loading. While the rest of all observed variables were retained at this stage as all were successfully loaded into their respective latent constructs. The results of CFA provided acceptable model fit indices and are presented in Table 2.

**TABLE 2:** Results of confirmatory factor analysis.

**Construct/Variable**	**Factor loadings**	**Alpha**	**CR**	**AVE**
Techno Stress		0.92	0.933	0.680
TS1	0.625			
TS2	0.718			
TS3	0.723			
TS4	0.773			
TS5	0.761			
TS6	0.767			
TS7	0.804			
TS8	0.815			
TS9	0.762			
TS10	0.771			
TS11	0.819			
TS12	0.804			
TS13	0.681			
Creative Self-efficacy		0.88	0.956	0.756
CE1	0.879			
CE2	0.848			
CE3	0.899			
CE4	0.861			
CE5	0.868			
CE6	0.855			
CE7	0.874			
Training		0.96	0.956	0.758
TR1	0.898			
TR2	0.860			
TR3	0.895			
TR4	0.868			
TR5	0.763			
TR6	0.862			
TR7	0.844			
Employee Performance		0.97	0.956	0.758
EP1	0.873			
EP2	0.866			
EP3	0.865			
EP4	0.880			
EP5	0.893			
EP6	0.826			
EP8	0.891			

*Goodness of fit Indices. χ^2^ = 751; d.f. = 521; χ^2^/d.f. = 1.44; *p* < 0.001; CFI = 0.97; GFI = 0.84; AGFI = 0.82; RMR = 0.06; RMSEA = 0.04.*

### Reliability and Validity

The reliability of scales used to measure latent constructs was assessed with the help of Cronbach’s alpha and composite reliability values. The results identified that values of both reliability measures were greater than the recommended cut-off value of 0.70 (Nunnally and Bernstein, 1994). Similarly, validity was assessed with the help of convergent validity and AVE values. For convergent validity, all observed variables were successfully loaded (having regression weights greater than 0.60) into their respective latent construct, and the AVE of all variables was greater than the proposed cut-off value of 0.5. Results of reliability and validity analysis are presented in Table 2.

Finally, the discriminant validity was assessed with the help of HTMT ratios. All constructs have HTMT ratios less than the cut-off value of 0.90 (Liang et al., 2007; Henseler et al., 2015). Hence, based on HTMT ratios, it was concluded that there was no issue of discriminant validity to report. HTMT ratios, shared variance, and correlation of latent constructs are presented in Table 3.

**TABLE 3:** Descriptive statistics and correlations.

	**Variable**	**No of items**	**Mean**	**s.d.**	**1**	**2**	**3**	**4**
**1**	TS	13	2.39	1.04	**0.68**			
**2**	CE	7	1.96	0.95	0.23* (0.07) ***0.27***	**0.75**		
**3**	TR	7	1.98	0.98	0.34* (0.12) ***0.36***	0.68*(0.46) ***0.73***	**0.76**	
**4**	EP	7	2.11	1.03	0.38* (0.14) ***0.39***	0.68*(0.46) ***0.84***	0.78*(0.61) ***0.88***	**0.76**

TS, Techno Stress; CE, Creative Self-efficacy; TR, Training; EP, Employee Performance; Shared variance in parenthesis; AVE in diagonal and bold; *P < 0.01; s.d.: Standard deviation; HTMT ratios in bold and italics.

### Hypotheses Testing

#### Stepwise Linear Regression Analysis

To test the first proposed hypothesis, stepwise linear regression in SPSS was used. Where in the first step, all demographic variables were added as control variables, and in the second step, technostress was entered. The results identified that technostress has a significant positive impact on employee performance in the presence of control variables. The results of stepwise linear regression analysis are presented in Table 4.

**TABLE 4:** Stepwise linear regression.

	DV: Employee Performance	
	**Standardized coefficient**	***t*-value**

**Step1 (Control Variables)**		
Gender	–0.235	–1.354
Age	0.068	1.063
Education	0.043	0.679
Experience	–0.045	–0.707
Job Status	0.155	1.097
University Sector	–0.231	–1.590
Discipline	–0.078	–1.149
**Step2 (Independent Variables)**		
Techno Stress	0.391*	6.257*
**Model Fit**		
*F*-value	6.158	
R2	0.19	
*p*-value	0.00	

*p < 0.01.

#### Moderation Analysis

PROCESS Macro (extension in SPSS) by Hayes (2013) was used to test the proposed moderation hypotheses for creative self-efficacy and training. PROCESS Macro by Hayes (2013) was preferred over simple regression analysis using interaction terms and structural equation modeling because of its robustness. PROCESS Macro uses a bootstrapping approach with biased corrected 95% confidence intervals and calculates the Johnson-Neyman outputs for the interaction term. The variables that define product terms were first mean-centered. Conditioning values at mean and ±1 SD and Johnson-Neyman outputs for the interaction graph were also calculated. Separate PROCESS Model No1 for creative self-efficacy and training as moderators were used. The results of PROCESS Model 1 are presented in Table 5.

**TABLE 5:** 5000 Bootstrap Results for PROCESS Model No.1, simple moderation analysis.

	**DV: EP**				**DV: EP**			
	**Estimate**	**SE**	**LL 95% CI**	**UL 95% CI**	**Estimate**	**SE**	**LL 95% CI**	**UL 95% CI**
TS	0.1483*	0.043	0.063	0.233	0.1074*	0.043	0.023	0.191
CE	0.7053*	0.052	0.602	0.809				
TS*CE	0.0881**	0.038	0.013	0.164				
TR					0.6981*	0.053	0.594	0.802
TS*TR					0.1141*	0.038	0.039	0.190
Model Fit								
*F*-value	122*				133*			
R2	0.62				0.64			
R2 Change	0.01**				0.02*			

TS, Techno Stress; CE, Creative Self-efficacy; TR, Training; EP, Employee Performance. *p < 0.01, **p < 0.05.

**FIGURE 2:**
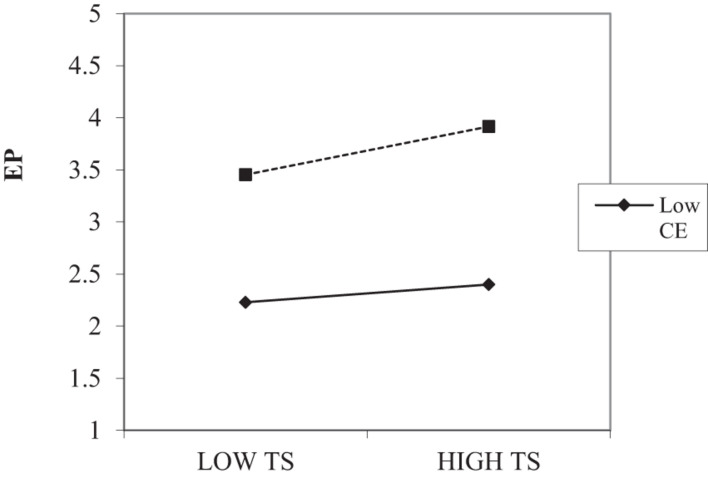
Technostress and Creative Self-efficacy Interaction Plot. TS, Techno Stress; CE, Creative Self-efficacy; EP, Employee Performance.

The results identified that the interaction terms for both CE and TR were significant, and there was no zero in the lower and upper bound of 95% confidence interval. An interaction graph for low and high (Mean ± SD) values of CE and TR were plotted. The interaction graph of TS and EP relationship (Shown in Figure 2) suggests that the relationship is significant for high levels of CE and insignificant for the low levels of CE. The slope test shows that the presence of CE enhances the positive relationship between TS and EP.

**FIGURE 3:**
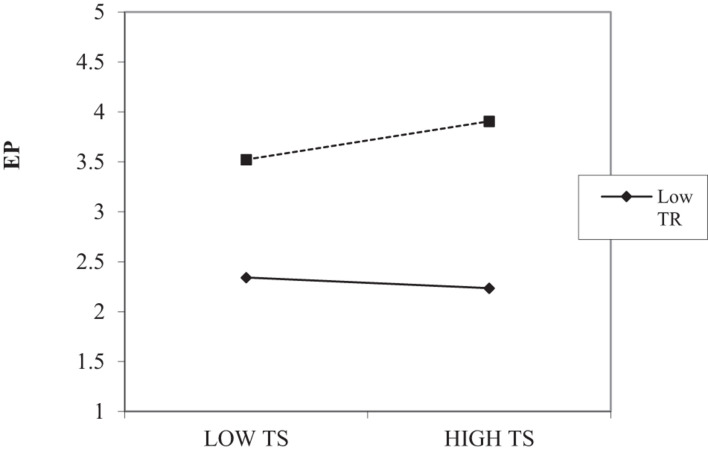
Techno stress and training interaction plot. TS, Techno Stress; TR, Training; EP, Employee Performance.

The interaction graph of TS and EP relationship (Shown in Figure 3) suggests that this relationship is significant for high levels of training and insignificant at low levels of training. The slope test shows that the presence of training enhances the positive impact of TS and EP.

Finally, to test the impact of both moderators simultaneously, PROCESS Macro Model No. 2 with 5000 bootstraps sampling and 95% biased corrected confidence intervals was used. The results of PROCESS Model no 2 are presented in Tables 6, 7. Code for Johnson-Neyman output for visualizing the interactions were also generated (Williams et al., 2003).

**TABLE 6:** 5000 Bootstrap Results for PROCESS Model No.2, moderation analysis with two moderators.

	**DV: EP**			
	**Estimate**	**SE**	**LL 95% CI**	**UL 95% CI**
TS	0.1059*	0.039	0.028	0.183
CE	0.3701*	0.061	0.249	0.491
TR	0.4380*	0.065	0.309	0.566
TS*CE	−0.0756*	0.045	–0.164	–0.013
TS*TR	0.1788*	0.044	0.091	0.266
**Model Fit**				
*F*-value	100*			
R2	0.70			
R2 Change	0.03*			

TS, Techno Stress; CE, Creative Self-efficacy; TR, Training; EP, Employee Performance. *p < 0.01.

**TABLE 7:** 5000 Bootstrap results for PROCESS Model No.2, test for higher-order unconditional interactions.

**Interaction**	**R2 Change**	***F*-Value**	***P*-value**
TS*CE	0.004	2.836	0.093
TS*TR	0.023	16.14	0.000
Both	0.024	8.46	0.000

*TS, Techno Stress; CE, Creative Self-efficacy; TR, Training.*

**FIGURE 4:**
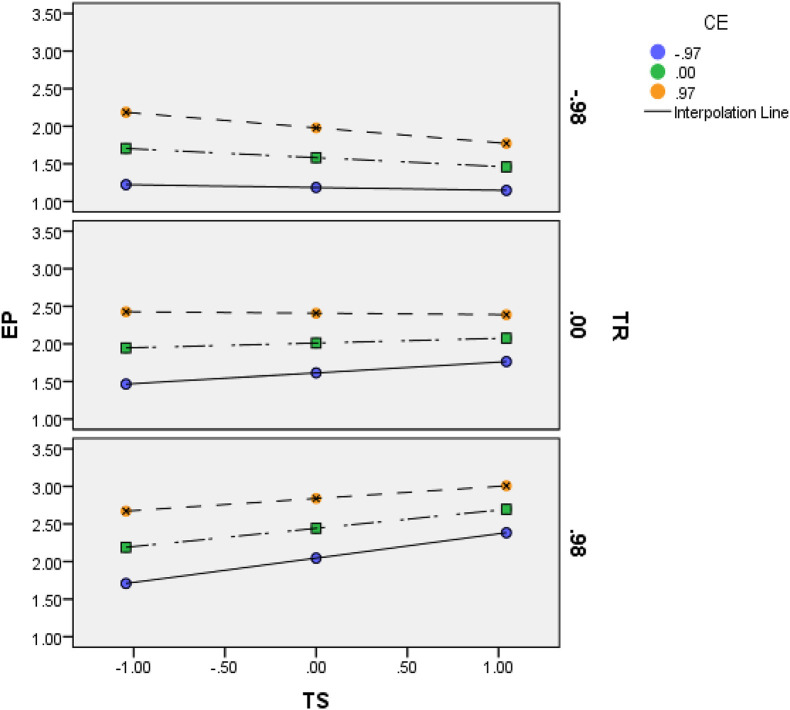
Three-Way Interaction Graph. TS, Techno Stress; CE, Creative Self-efficacy; TR, Training; EP, Employee Performance.

The graph for three-way interaction identified that at a high level of TR and CE the relationship of TS and EP becomes significant. Or in other words, at high levels of TR, CE, and TS, employees’ performance increases. While at a low level of TR and high level of CE and TS the EP decreases the three-way interaction graph is presented in Figure 4.

## Discussion

The current investigation was focused on studying the impact of technostress that arose due to intensive technology use during COVID-19 lockdown on university instructors’ performance. The moderating impact of training and creative self-efficacy on the technostress and performance relationship were also investigated. The most important finding is related to the positive impact of technostress on instructor’s performance. This study was conducted during the COVID-19 lockdown period when the university instructors were confined to their homes. For the first time in Pakistan, university education was forced to transfer wholly online, and the instructors’ were not in direct contact with students. Technology brings ease of work that saves energy and time. For this reason, instead of taking technostress as a distress factor university instructors considered it as eustress (Tarafdar et al., 2019). ICT is revolutionizing learning and defining the organizational structure of higher education (Ortagus et al., 2018). Undoubtedly, ICT brings unprecedented benefits to higher education, such as enlarging and democratizing learners’ access to quality educational resources and providing learners with convenience and personalized learning experiences. Technostress inhibitors are described as available facilitating resources (e.g., knowledge and support) that could decrease negative consequences caused by technostress creators and improve productivity and performance (Tarafdar et al., 2010; Fuglseth and Sørebø, 2014).

The results revealed support for the first hypothesis highlight that digital technologies can positively affect employee performance. The current study results are aligned with the studies showing a positive relationship between technology use and performance (Tarafdar et al., 2010, 2015). Dragano and Lunau (2020) found the technology to positively affect employee’s occupational health and well-being that encourage them to concentrate on their work. The same results were revealed by the earlier studies that ICT can ensure better work management and is associated with improved employee well-being (Ter Hoeven and Van Zoonen, 2015). Similarly, the effects of automation were examined in the pharmacy, where the technology reduced the stress (James et al., 2013). The use of technology provides opportunities like easy access to help desk and technical support to address problems university instructors encounter in their work could ease their stressed feelings and improve their performance (Skaalvik, et al., 2017). Also, the involvement of university instructors in planning, implementation, assessment, and refinement phases of ICT integration in higher education may diminish their technostress by considering university instructors’ actual needs and ICT requirements for their work (Tarafdar et al., 2010; Califf and Brooks, 2020).

The second hypothesis related to the moderating effect of training on technostress and employee performance linkage is also accepted. The training is the transfer of knowledge and skills to develop abilities. The training is vital to coping up with environmental changes like the COVID-19 outbreak. It enables the employees to solve their work-related problems (Athar and Shah, 2015) and adds to their confidence to do work (Caldwell et al., 2009; Aziz, 2015). The training inculcates time management and work management skills among employees (Aziz, 2015) and reduces their stress levels to achieve the performance targets. Moreover, the training reduces technostress by improved attitude and behaviors developed. These improved attitudes and behaviors simplify the work processes, thus reduced errors and rework (Elnaga and Imran, 2013). The acquired training keeps employees calm during workload hours and enables them to work efficiently and effectively. The IT training empowers employees with reduced problems and enhanced learning services delivery (Itzchakov, 2020). The availability of training and other helping material encouraged university staff to learn more about the technology, thus reducing technostress. They started conveniently using technology and managed to perform as per the schedules.

The third hypothesis related to the moderating effects of creative self-efficacy on the relationship between technostress and employee performance is also accepted. Creativity leads to newer ways to tackle assigned tasks and confidence to perform well (Elnaga and Imran, 2013; Ismail et al., 2019). Creative employees believe in competitiveness. They share their innovative ideas to produce effective shortcuts and solutions to the problems (Kremer et al., 2019). Problem-solving helps in saving time and resources. The creative people devise more innovative solutions (Cheng, 2019), resulting in continued working and sustainability. One’s creative efficacy enables him/her to show perseverance in the time of crisis like work overload during COVID-19 that also enhance their performance. Similarly, when training and combined with creative self-efficacy the results are more promising. Training also enhances creativity among employees (Holman et al., 2020), and employees with creative skills provide support to other employees entangled with technology problems. The creativity and their creative efficacy, as a resource, bring them better performance.

### Theoretical Implications

The current investigation offers few important theoretical implications. First, the relationships were discussed in the light of social exchange theory (Ekeh, 1974; Cook et al., 2013). The SET and transactional model of stress and coping were generalized (Lazarus and Folkman, 1984) in a developing country, Pakistan context during the COVID-19 time. The proposed model in the study contributes by going beyond the stress and strain mitigation process that prior technostress studies have focused on (Ragu-Nathan et al., 2008; Tarafdar et al., 2011, 2015; Fuglseth and Sørebø, 2014; Galluch et al., 2015; Salo et al., 2017). The study has not only looked into the user’s own way of mitigating the technostress by using creative self-efficacy but have also taken in account the external help for mitigating the technostress by considering the training provided by organizations.

Secondly, with this study, it is explained that how creative self-efficacy and training reduce technostress and enhance employee performance as a social exchange tool. Generally, the organization provides training to its employees, which is considered a means of support. In return, the employees feel valued. As a reciprocal behavior, they try to show exceptional performance. Current investigation confirms the prior literature highlighting that whenever there is a give and take situation, both parties try to offset the favor given by the other party. The social exchange theory (Ekeh, 1974; Cook et al., 2013) provides an alternative explanation for describing employees’ performance through various factors. Training and creative self-efficacy are important mechanisms that help reduce the adverse effects of technostress and help in enhancing performance. In organizations, the individuals interact and develop relationships that are strengthened by developing a sense of trust and reciprocity. This trust and sense of reciprocity motivate employees to perform better to fulfill the organizational goals (Cook et al., 2013). Such exchanges can potentially reduce stress levels, enhance motivation, and gives confidence to employees to perform better.

Lastly, the current investigation has also generalized and extended the transactional model of stress and coping by identifying two important coping mechanisms to reduce the technostress, including the creative self-efficacy triggered from inside by the individual and training and development triggered from outside by the organization. It is important to note that coping strategies by an individual are not only the outcome of an individual’s own capabilities, they can be a result of external help, too, in the form of training provided by organizations.

### Practical Implications

There are few important practical impactions to report too. Undoubtedly, ICT brings unprecedented benefits to higher education, such as enlarging and democratizing learners’ access to quality educational resources and providing learners with convenient and personalized learning experiences. Technostress inhibitors are described as available facilitating resources (e.g., knowledge and support) that could decrease negative consequences caused by technostress creators and improve productivity and performance (Tarafdar et al., 2010; Fuglseth and Sørebø, 2014). The use of technology provides opportunities like easy access to help desk and technical support to address problems university instructors encounter in their work could ease their stressed feelings and improve their performance (Skaalvik et al., 2017).

The use of technology encouraged instructors to have more energy to work, and thus their performance was enhanced. The different applications like the alarm systems and reminders helped employees prioritize and remember what to do. Sharing data with the students became easy by taking pictures or making videos, and sharing it with students helped them share the exact information without interruption. The use of technology helped instructors take more time off than they spent with their families and enjoyed better work-family balance.

University instructors might feel insecure about their job due to the fear of being replaced by new learning and teaching technologies or other people with higher ICT capabilities (Califf and Brooks, 2020). After adequate training on how to use technology, they can overcome the stress of the fear to be replaced. Hence, for the management of universities, it is important to provide adequate training to the instructors so their performance is not affected negatively due to technostress. Similarly, as an important practical implication, the university administrators must allocate the required resources and provide support to the employees for their improved performance. The university managers can establish work standards aligned with the prevailing environmental conditions. It will help instructors to avoid the negative consequences of technostress and conveniently perform their work. Moreover, the university administration must adopt strategies like providing appropriate training and help in developing creative self-efficacy to minimize the harmful effects of technostress among university staff.

The university administration and instructors must consider technology as a positive tool for performance and should take training as a helping tool for improving deficient skills. Keeping in view the current study’s findings, it is suggested that the technology can be used in every sphere of university functioning to boost performance. Increasing the individual’s work capacity can help instructors to perform without errors and on time. Enhancing information systems-related knowledge and skills will not only add to an individual’s confidence to work but also adds to his/her creative self-efficacy. Creative self-efficacy sufficiently minimizes technostress and enhances the performance of individuals (Pirkkalainen et al., 2019). The managers must adopt strategies to enhance the creative self-efficacy of employees to ensure higher proficiency. It is also observed that work from home also adds to the creative self-efficacy of university employees. The overloaded people look for ways of intelligent use of technology (Sintema, 2020). The use of recorded lectures and other options helped them to manage their performance. They learned how to manage time and energy to work more creatively and were able to perform better. The employees who managed time and their work were better able to relieve stress, and again this supported them to concentrate on work, thus enhancing their performance (Asaloei et al., 2020). Apart from training, they tend to learn from various sources that give them the confidence to work without mistakes and deliver quality material. It also adds to their satisfaction and leads to a higher performance level. Lastly, it is important for university administration to pose technology as an opportunity instead of a challenge or a threat to employees. Similarly, separating performers from non-performers of technology-intensive tools can also help in identifying individuals with the need for training.

## Limitations and Future Directions

This study also has few limitations like other research studies. Firstly, the study was conducted using questionnaires as a single source of gathering responses. It may result in common method bias. This bias can be controlled by the simultaneous use of multiple data collection sources (Podsakoff et al., 2003; Schwarz et al., 2017). The present study was a cross-sectional study focused on a single set of employees that are university instructors. The longitudinal study design using the same framework may bring in additional interesting insights. The addition of other constructs to the framework will also be a healthy contribution to the existing framework, such as the employee’s work-life balance, the quality of work-life and the personality traits, etc.

Moreover, organizational factors, such as organizational support, may also strengthen the existing framework. The present study included the employees’ performance and looked mainly at the task performance of employees. The technostress may also affect other dimensions of employee performance, such as contextual or adaptive performance. It is also possible that the other organizational supports other than training only may also add to the current framework. Lastly, it is recommended that future studies can empirically test the reciprocity (Li et al., 2014) between the organization and the employees regarding coping strategies based on the social exchange theory as well as they can also extend the proposed model by including the primary appraisal stage of transactional model of stress and coping.

## Conclusion

The phenomenon of technostress is emerging and needs much examination, especially during COVID-19 lockdown. Technology is seen as an opportunity rather than a threat to working better by the university faculty members. Instead of the distress, the technostress worked as eustress for university instructors who had to convert their face-to-face teaching to online during COVID-19 lockdown. Additionally, the finding identified training and creative self-efficacy as important facilitating mechanisms. Hence, appropriate training and making the best use of employees’ creative self-efficacy benefited the employees to cope with technostress and performance issues. The paper theoretically and empirically extends the current literature on technostress and performance by highlighting that performance is positively affected by technology instead of adverse effects.

## Data Availability Statement

The raw data supporting the conclusions of this article will be made available by the authors, without undue reservation.

## Author Contributions

FS revised the manuscript as per the suggestions of the reviewers and revised data analysis, and wrote up of the results. MM upgraded all the revisions regarding introduction and literature review. SSQ revised the methodology section and results as per the suggestions of the reviewers. MF revised the discussion and conclusion section. SQ revised the methodology and conclusion of the manuscript. All authors contributed to the article and approved the submitted version.

## Conflict of Interest

The authors declare that the research was conducted in the absence of any commercial or financial relationships that could be construed as a potential conflict of interest.

## Publisher’s Note

All claims expressed in this article are solely those of the authors and do not necessarily represent those of their affiliated organizations, or those of the publisher, the editors and the reviewers. Any product that may be evaluated in this article, or claim that may be made by its manufacturer, is not guaranteed or endorsed by the publisher.

## Annexure

**Scales used for measurement of variables**

1- Techno-Stress adapted from Tarafdar et al. (2007)

1. Techno Overload
2. I am forced by social media and other computer-related technologies used for teaching and other work -related purposes to work much faster
3. I am forced by computer-related technologies used for teaching and other work -related purposes to do more work than I can handle.
4. I am forced by social media and other computer-related technologies used for teaching and other work -related purposes to work with very tight time schedules.
5. I am forced to change my work habits to adapt to new computer-related technologies used for teaching and other work -related purposes.
6. I have a higher workload because of increased computer-related technologies used for teaching and other work -related purposes complexity (flow of Information).
7. Techno-invasion
8. I spend less time with my family due to computer-related technologies used for teaching and other work -related purposes used for teaching and other work related purposes.
9. I have to be in touch with my work even during my vacation due to social media and other computer-related technologies used for teaching and other work -related purposes.
10. I have to sacrifice my vacation and weekend time to keep current on new technologies.
11. I feel my personal life is being invaded by computer-related technologies used for teaching and other work -related purposes.
12. Techno-complexity
13. I do not know enough about computer-related technologies used for teaching and other work -related purposes to handle my job satisfactorily.
14. I need a long time to understand and use new computer-related technologies used for teaching and other work -related purposes.
15. I do not find enough time to study and upgrade my computer-related technologies skills used for teaching and other work -related purposes.
16. I often find it too complex for me to understand and use new computer-related technologies used for teaching and other work -related purposes.
17. I find new recruits to this organization know more about computer-related technologies used for teaching and other work -related purposes than I do.

2- Creative self-efficacy adapted from Brockhus et al. (2014)

1. I am a creative person
2. I can solve problems efficiently even complicated problems
3. I trust into my creative abilities
4. Compared to my colleagues my ideas are outstanding
5. I can deal with problems requiring creative thinking
6. I am good in proposing “out of the box” solutions
7. I am confident that I can develop creative solutions for almost any problem

3- Training adapted from Aziz (2015).

1. I can list down all the important things emphasized in this training
2. I know how to solve certain job problems using the skills taught in this training
3. I know how to work more efficient using the knowledge learned in this training
4. I have the capability to perform the skills taught in this training
5. My personal competencies have improved after attending this training
6. I am being more professional in certain tasks after attending this training
7. My job performance has improved as a result of applying the skills emphasized in this training

4- Performance adopted from Koopmans et al. (2014)

1. In the past three months I took on extra responsibility
2. In the past three months I started new tasks myself, when my old ones were finished
3. In the past three months I took on challenging work tasks, when available
4. In the past three months I worked at keeping my job knowledge up –to –date
5. In the past three months I worked at keeping my job skills up-to-date
6. In the past three months I came up with creative solutions to new problems.
7. In the past three months I kept looking for new challenges in my job
8. In the past three months I actively participated in work meetings
